# Emicizumab prophylaxis in a preterm infant with severe hemophilia A: a case report on the feasibility of early use

**DOI:** 10.1016/j.rpth.2026.103401

**Published:** 2026-02-28

**Authors:** Eman Hassan, Charles Percy, Amna Ahmed, Gillian Lowe, Will Lester, Neil V. Morgan, Jayashree Motwani

**Affiliations:** 1 Department of Cardiovascular Sciences, College of Medicine and Health, University of Birmingham, Birmingham, United Kingdom; 2 Department of Haematology, Queen Elizabeth Hospital, Birmingham, United Kingdom; 3 Department of Haematology, Birmingham Children’s Hospital, Birmingham, United Kingdom

**Keywords:** emicizumab, hemophilia A, intracranial hemorrhages, low-birth-weight, preterm, prophylaxis

## Abstract

**Background:**

Emicizumab provides effective prophylaxis for hemophilia A (HA), but evidence for preterm and very low-birth-weight infants remains limited.

**Key Clinical Question:**

Can emicizumab be safely initiated shortly after birth in a preterm, low-birth-weight infant with severe HA?

**Clinical Approach:**

We report a male infant born at 30 + 4 weeks of gestation to a known carrier of severe HA. Because of the high risk of intracranial hemorrhage, the case was reviewed by a multidisciplinary team with shared decision-making. After confirming severe HA and excluding intracranial hemorrhage on cranial ultrasonography, emicizumab prophylaxis was started at 26 hours of life, at a weight of 1.48 kg. Monitoring included emicizumab levels, thrombin generation, and inhibitor testing. The infant completed the loading phase and transitioned to maintenance dosing without bleeding, thrombosis, or adverse events.

**Conclusion:**

This case supports the feasibility and early safety of initiating emicizumab in preterm, low-birth-weight infants.

## Essentials

• Emicizumab prevents bleeding in HA, but data on preterm babies are scarce.
• This report describes emicizumab use at birth to reduce intracranial bleeding risk in a preterm infant with HA.
• Early use was safe, with no bleeding or complications during follow-up.
• Findings suggest emicizumab may be feasible for low birth-weight, preterm infants.

## 1 Introduction

Emicizumab (Hemlibra) is the first approved and commercially available nonfactor replacement for people with hemophilia A (HA) of all ages, with and without factor (F)VIII inhibitors [1,2]. This recombinant, bispecific, monoclonal antibody bridges activated FIX and FX, mimicking the cofactor function of activated FVIII [3]. The subcutaneous administration of emicizumab provides sustained pharmacokinetics (PK) and allows prophylaxis to begin at a very young age. This can reduce bleeding episodes and life-threatening intracranial hemorrhage (ICH) while avoiding complications related to central venous access, decreasing treatment burden, and improving adherence [4]. A key advantage of early emicizumab prophylaxis is its potential to defer or avoid FVIII exposure, thereby decreasing the risk of inhibitor development in previously untreated patients [4]. While clinical trials and real-world data have established the safety and efficacy of emicizumab in infancy and early childhood, reports in preterm neonates are exceptionally limited, and evidence to guide its use in this vulnerable group remains lacking [5]. Herein, we report a case of emicizumab use in a preterm, 30-week-gestation, low-birth-weight infant with severe HA, illustrating the potential feasibility of early prophylaxis in this high-risk population.

## 2 Case Presentation

The patient was born to a known carrier of HA, with 2 older male siblings also affected by HA. Both siblings were initially managed with recombinant FVIII (rFVIII) prophylaxis and later switched to emicizumab, achieving excellent bleeding control. There was no family history of FVIII inhibitors.

The pregnancy was unplanned and proceeded without preimplantation genetic testing. In line with maternal preference, no invasive prenatal diagnostic procedures were performed. Early in the pregnancy, discussions were held with the family regarding management options should the baby be affected. The parents were keen to initiate emicizumab prophylaxis, given their positive experience with the older siblings.

At 29 weeks of gestation, the mother developed premature contractions. As preterm delivery became likely, the parents were counseled regarding the substantially increased risk of ICH in premature neonates and the anticipated need for prophylaxis from birth. The potential requirement for rFVIII in the event of bleeding or ICH was discussed, and both prophylactic options, rFVIII and emicizumab, were reviewed in detail, including the limited evidence for emicizumab use in preterm and low-birth-weight infants. It was also explained that hemostatic protection during the initial emicizumab loading phase is variable and cannot be precisely predicted. Despite these uncertainties, the parents remained strongly in favor of emicizumab to avoid central venous access and its associated risks.

Delivery occurred spontaneously at 30 weeks + 4 days of gestation via an uncomplicated normal vaginal delivery. The male neonate weighed 1.6 kg, had no congenital anomalies or dysmorphic features, and received a single standard dose of intravenous vitamin K at birth. A venous blood sample confirmed severe HA, with FVIII < 1 IU/dL. The baseline activated partial thromboplastin time was >100 seconds. Due to limitations in the sampling volume, baseline thrombin generation (TG) was not performed prior to treatment initiation. Due to prematurity, the infant was admitted to the neonatal intensive care unit for monitoring. A multidisciplinary meeting was held to determine the optimal timing for prophylaxis initiation, using a shared decision-making approach with the parents. Although emicizumab was planned for early initiation, exclusion of ICH was required before treatment. After cranial ultrasonography confirmed no ICH and the infant remained clinically stable without bleeding, emicizumab was commenced at 26 hours of life. Loading dose of 3 mg/kg weekly for 4 weeks, calculated based on the infant’s weight at this point, was 1.48 kg. To ensure accurate administration of the very small emicizumab doses, 1 mL neonatal low-dead-space syringes were used.

Given the constraints on frequent blood sampling, emicizumab levels and associated laboratory monitoring were performed at week 1 (prior to the second dose), after completing the loading phase, and at week 13 to monitor the maintenance dose. Assessments included TG, emicizumab plasma concentration, FVIII activity, and inhibitor testing. Weight was recorded before each weekly dose. The relevant clinical and laboratory results are summarized in the Table. Progressive improvement in TG was observed over time, with week 13 values within the normal range. The Figure illustrates this trend, showing improved TG parameters with increasing emicizumab plasma concentrations.

**Table:** Clinical and laboratory investigations at different time points throughout emicizumab prophylaxis.

Time point	Weight (kg)	Emicizumab level (μg/mL)	aPTT (s)	Lag time (min)	ETP (nM.min)	Peak TG (nM)
Wk 1	1.6	11	31	**7.7**	**569**	**34.9**
Wk 5 (after loading)	2.27	23	31	**6.1**	653	**31.9**
Wk 13	4	30	28	4.55	816	58.9

Normal ranges: emicizumab (40-60 μg/mL); aPTT (25-34 seconds); mean TG includes lag time (2.8-5.1 minutes), ETP (580-2277 nM.min), and peak TG (42-229 nM). Due to the nature of the study and ethical constraints on recruiting age-matched neonatal controls, reference ranges were derived from 40 healthy adult controls using mean ± 2 SD (age range, 16-73 years). TG results outside control ranges are indicated in bold.

aPTT, activated partial thromboplastin time; ETP, endogenous thrombin potential; TG, thrombin generation.

**Figure:**
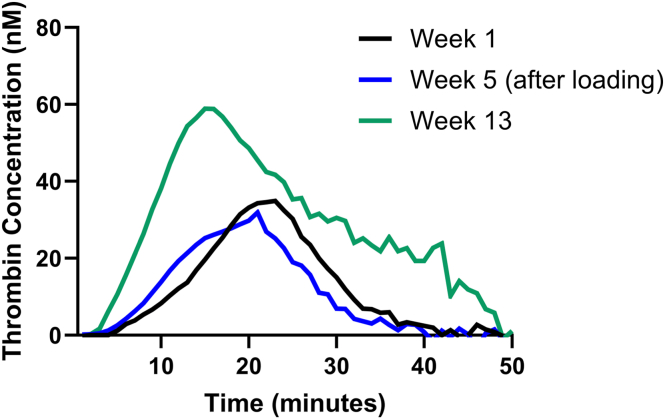
Calibrated automated thrombogram results showing improvement in thrombin generation (TG) parameters over time. TG curves for each of the 3 measurements (week 1, black; week 5 (after loading), blue; week 13, green) after stimulation with tissue factor in PRP Reagent (670 nM). TG was measured using the following reagents: platelet-rich plasma Reagent, thrombin calibrator, and fluorescent substrate/CaCl2 (FluCa, Stago).

FIX and FX levels were measured at week 1, with values of 38 U/dL and 63 U/dL, respectively. Genetic testing confirmed the pathogenic mutation causing severe HA, c.5406T>G p.(Tyr1802∗), identical to that identified in the mother and siblings.

Serial weekly cranial ultrasounds showed no ICH. The infant required brief oxygen support, was weaned successfully, and was discharged after 4 weeks.

Maintenance emicizumab dosing continued at 3 mg/kg administered every 2 weeks, adjusted for weight gain. Fortnightly reviews were arranged to monitor weight and ensure accurate dosing. Routine immunizations were administered subcutaneously without complications. At 4 months of corrected gestational age, the patient had an inguinal hernia repair procedure and did not require additional hemostatic support, apart from tranexamic acid. The procedure was uneventful with no peri- or postoperative bleeding. At 6 months of age, the patient remained clinically stable, had experienced no spontaneous or traumatic bleeding episodes, and had not received treatment with FVIII. FVIII inhibitor screen remains negative. To date, no adverse events or injection-site reactions have been reported in this case.

Written informed consent for blood collection was obtained from the patient’s parent (the mother). Blood samples were collected via the Human Biomaterials Resource Centre, University of Birmingham, under National Health Service Research Ethics Committee approval (North West Haydock Research Ethics Committee, reference 25/NW/0013) and Human Biomaterials Resource Centre application number 20-369.

## 3 Discussion

In this case, emicizumab was started at 26 hours of life in a 30-week-gestation infant weighing 1.48 kg, representing 1 of the earliest emicizumab uses documented in the literature. The rationale to proceed with emicizumab prophylaxis was defined by several key elements: prematurity, increased ICH risk, and the parents’ well-informed consent after counseling about both FVIII and emicizumab. Preterm infants are inherently predisposed to ICH due to germinal matrix fragility and immature cerebrovascular autoregulation, with up to 20% of very preterm neonates affected [6]. The overall incidence of ICH in infants with hemophilia is estimated at 2.1% in the neonatal period (95% CI, 1.5%-2.8%); consequently, the coexistence of prematurity and hemophilia substantially amplifies this risk [7,8]. In neonates with severe HA, the absence of prophylaxis confers a 33-fold higher risk of life-threatening ICH compared with infants without hemophilia, often resulting in irreversible neurological sequelae [7,8]. Therefore, initiating prophylaxis very early in life, especially in premature infants, should be considered the standard of care.

Premature infants differ markedly in physiology compared with term neonates and have distinct hemostatic profiles. Traditional FVIII replacement in preterm infants is challenging due to the need for intravenous access and unpredictable PK. FVIII replacement often requires more frequent dosing to maintain adequate trough levels, increasing cumulative exposure and inhibitor risk, an effect possibly amplified by the immature immune response and systemic stress seen in prematurity [9,10]. Early emicizumab prophylaxis can limit FVIII exposure during this vulnerable period, potentially reducing inhibitor development. There have been theoretical concerns about reduced efficacy of emicizumab during the first months of life due to physiologically low FIX levels; however, FIX levels in this case were within the normal postnatal range.

Evidence supporting the use of emicizumab in premature infants remains limited. However, data from major clinical studies reinforce its safety and efficacy from birth. The Hemophilia A Study Evaluating Prophylaxis With Emicizumab (HAVEN) 7 trial demonstrated that infants as young as 9 days old can be safely treated, with no ICH events reported [4]. Real-world data further support these findings, showing complete bleed prevention and absence of inhibitor development in infants treated within the first 2 months of life [11]. The ongoing Phase III Study of Emicizumab Prophylaxis in Persons With Hemophilia A in Japan (HINODE) study is now extending this evaluation to infants under 12 months, integrating TG and coagulation potential analyses to establish reference data for physiological hemostasis in early infancy [12]. Although most participants in these studies were term or late preterm, the collective evidence supports early initiation of emicizumab when clinically indicated.

The Medical and Scientific Advisory Council of the National Bleeding Disorders Foundation and National Bleeding Disorders Foundation recommendations emphasize individualized risk evaluation when initiating prophylaxis, particularly before emicizumab reaches steady-state after 4 weekly loading doses [13,14]. In this case, the treating team mitigated risk by closely monitoring PK parameters, including plasma emicizumab levels, activated partial thromboplastin time, TG, and weekly cranial ultrasonography. Although emicizumab concentrations and TG parameters showed progressive improvement and normalized by week 13, the comparatively lower values observed after the loading phase were below those typically reported in older children. The week 1 measurements should be interpreted cautiously, as results after a single dose do not reflect steady-state PK. The early attenuation in emicizumab levels and TG may reflect prematurity, low-birth-weight, or age-related physiological differences influencing emicizumab exposure. While dosing adjustments cannot be concluded, these findings suggest that standard weight-based dosing may not fully account for PK variability in low-birth-weight neonates. Further evaluation of emicizumab exposure and dosing strategies in this population is warranted.

Overall, the laboratory results were satisfactory, with no clinical evidence of bleeding, thrombosis, or other adverse events, highlighting emicizumab’s favorable tolerability in preterm infants. Notably, the most common adverse effect, minor injection-site reactions reported in approximately 16% of participants in the HAVEN 7 trial, was not observed in this case [4].

Additional evidence is needed to clarify the long-term safety of emicizumab in preterm and low-birth-weight infants. Pending such evidence, treatment decisions should rely on individualized clinical assessment, supported by multidisciplinary review and shared decision-making.

## References

[1] HEMLIBRA® (2017). https://www.gene.com/download/pdf/hemlibra_prescribing.pdf.

[2] (2018). European Medicines Agency. Hemlibra (emicizumab): EPAR – product information.

[3] Gelbenegger G., Schoergenhofer C., Knoebl P., Jilma B. (2020). Bridging the missing link with emicizumab: a bispecific antibody for treatment of hemophilia A. Thromb Haemost.

[4] Pipe S.W., Collins P., Dhalluin C., Kenet G., Schmitt C., Buri M. (2024). Emicizumab prophylaxis in infants with hemophilia A (HAVEN 7): primary analysis of a phase 3b open-label trial. Blood.

[5] Young G., Pipe S.W., Kenet G., Oldenburg J., Safavi M., Czirok T. (2024). Emicizumab is well tolerated and effective in people with congenital hemophilia A regardless of age, severity of disease, or inhibitor status: a scoping review. Res Pract Thromb Haemost.

[6] Zhou M., Wang S., Zhang T., Duan S., Wang H. (2024). Neurodevelopmental outcomes in preterm or low birth weight infants with germinal matrix-intraventricular hemorrhage: a meta-analysis. Pediatr Res.

[7] Zwagemaker A.F., Gouw S.C., Jansen J.S., Vuong C., Coppens M., Hu Q. (2021). Incidence and mortality rates of intracranial hemorrhage in hemophilia: a systematic review and meta-analysis. Blood.

[8] Singleton T.C., Keane M. (2012). Diagnostic and therapeutic challenges of intracranial hemorrhage in neonates with congenital hemophilia: a case report and review. Ochsner J.

[9] Chalmers E.A., Brown S.A., Keeling D., Liesner R., Richards M., Stirling D. (2007). Early factor VIII exposure and subsequent inhibitor development in children with severe haemophilia A. Haemophilia.

[10] Moorehead P.C., Chan A.K.C., Lemyre B., Winikoff R., Scott H., Hawes S.A. (2018). A practical guide to the management of the fetus and newborn with hemophilia. Clin Appl Thromb Hemost.

[11] Ahmed S., Kavanagh M., Kelly I., Ferry C., Mullen B., Brady B. (2024). Safety and outcome of early start of emicizumab in neonates and infants with severe hemophilia α, a real-world experience. Blood.

[12] Ohga S., Takeyama M., Ishimura M., Inoue H., Nosaka D., Iwasaki K. (2024). HINODE study: haemophilia A in infancy and newborns – protocol for a prospective, multicentre, observational study evaluating the coagulation potential and safety of emicizumab prophylaxis. BMJ Open.

[13] National Hemophilia Foundation (2020). https://www.bleeding.org/sites/default/files/document/files/258_emicizumab.pdf.

[14] National Bleeding Disorders Foundation (NBDF) (2022). MASAC document 268: Recommendation on the use and management of emicizumab-kxwh (Hemlibra®) for hemophilia A with and without inhibitors. https://www.bleeding.org/healthcare-professionals/guidelines-on-care/masac-documents/masac-document-268-recommendation-on-the-use-and-management-of-emicizumab-kxwh-hemlibrar-for-hemophilia-a-with-and-without-inhibitors.

